# Supplementary material for: A Highly Sensitive SERS and RRS Coupled Di-Mode Method for CO Detection Using Nanogolds as Catalysts and Bifunctional Probes
Source: Nanomaterials (Basel). 2020 Mar 2;10(3):450. doi: 10.3390/nano10030450 (PMC7153473; doi:10.3390/nano10030450)
Supplement: Supplementary file 1 [file nanomaterials-10-00450-s001.pdf]

A Highly Sensitive SERS and RRS Coupled Di-Mode Method for CO Detection Using Nanogolds as Catalysts and Bifunctional Probes

Dongmei Yao [1,2,3], Guiqing Wen [1,2] , Lingbo Gong [1,2], Chongning Li [1,2], Aihui Liang [1,2,*] and Zhiliang Jiang [1,2,*]

1 Key Laboratory of Ecology of Rare and Endangered Species and Environmental Protection (Guangxi Normal University), Ministry of Education, Guilin 541004, China; dmyao47@163.com (D. Y.); gqwen@mailbox.gxnu.edu.cn (G. W.); zljiang89@126.com (L. G.); lcn7882342@163.com (C. L.)
2 Guangxi Key Laboratory of Environmental Pollution Control Theory and Technology for Science and Education Combined with Science and Technology Innovation Base, Guilin 541004, China
3 College of Chemistry and Biology Engineering, Hechi University, Yizhou, 546300, China
* Correspondence: zljiang@mailbox.gxnu.edu.cn (Z. J.); ahliang2008@163.com (A. L.)

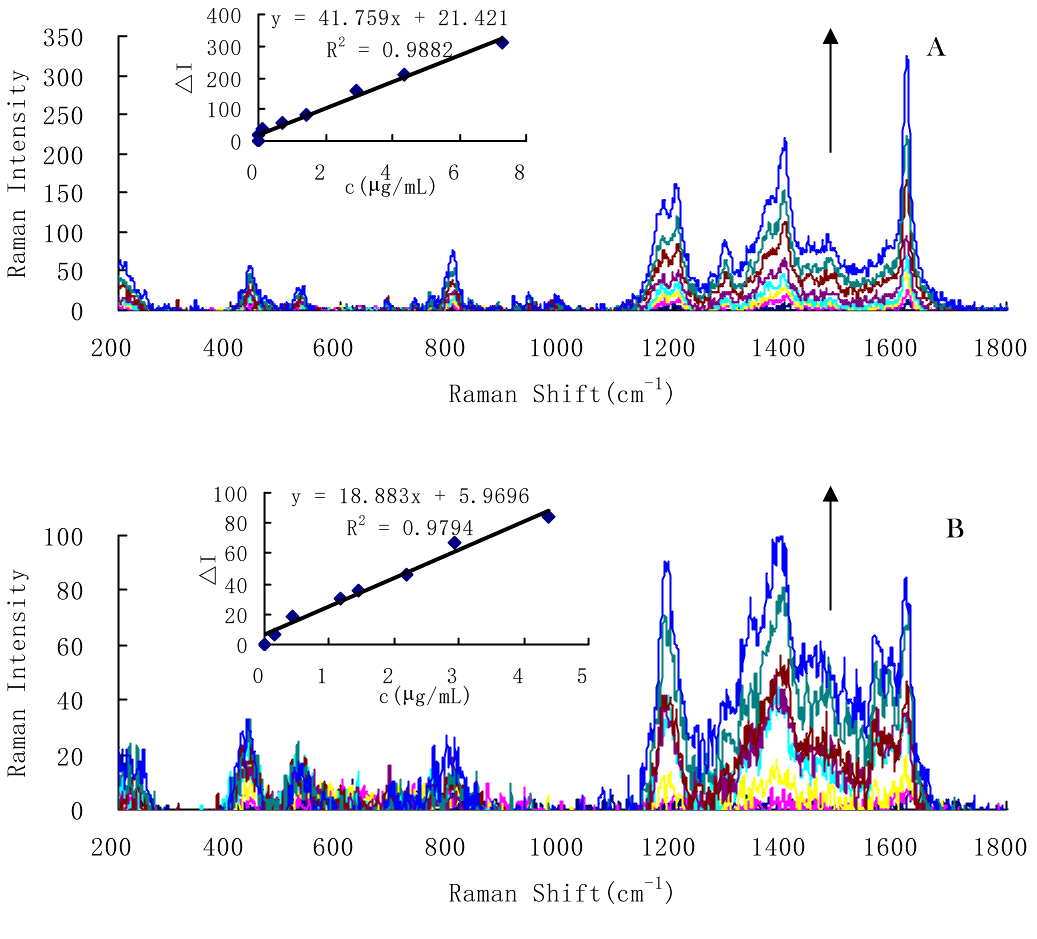

Figure S1. SERS spectra. A: CO-$HAuCl_4$- citrate system. a: 0.24 mmol/L pH 5.2 HAc-NaAc+5.6 mmol/L citrate+0.61 mmol/L HCl+56.8 μg/mL $HAuCl_4$+0.76 μmol/L VBB+5.9 mmol/L NaCl; b: a+0.03 μg/mL CO; c: a+0.07 μg/mL CO; d: a+0.15 μg/mL CO; e: a+0.73 μg/mL CO; f: a+1.5 μg/mL CO; g: a+2.2 μg/mL CO. B: CO-$HAuCl_4$-glucose system. a: 0.24 mmol/L pH 5.2 HAc-NaAc+5.6 mmol/Lglucose+0.56 mmol/L HCl+56.2 μg/mL $HAuCl_4$+0.76 μmol/L VBB+5.9 mmol/L NaCl; b: a+0.1 μg/mL CO; c: a+0.4 μg/mL CO; d: a+1.2 μg/mL CO; e: a+1.5 μg/mL CO;

f: a+2.2 μg/mL CO; g: a+2.9 μg/mL CO; h: a+4.4 μg/mL CO.

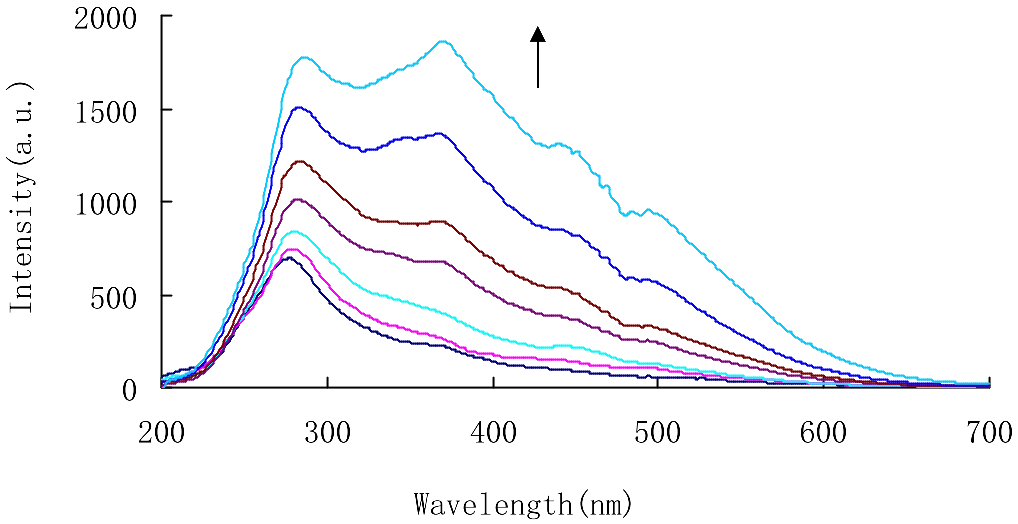

Figure S2. The RRS spectra of CO-$PdCl_2$ system. (a)pH 5.2 HAc-NaAc+7.5 μg/mL $PdCl_2$; (b) a+0.1 μg/mL CO; (c)a+0.2 μg/mL CO; (d)a+0.6 μg/mL CO; (e)a+0.75 μg/mL CO; (f) a+1.25 μg/mL CO; (g)a+2.0 μg/mL CO.

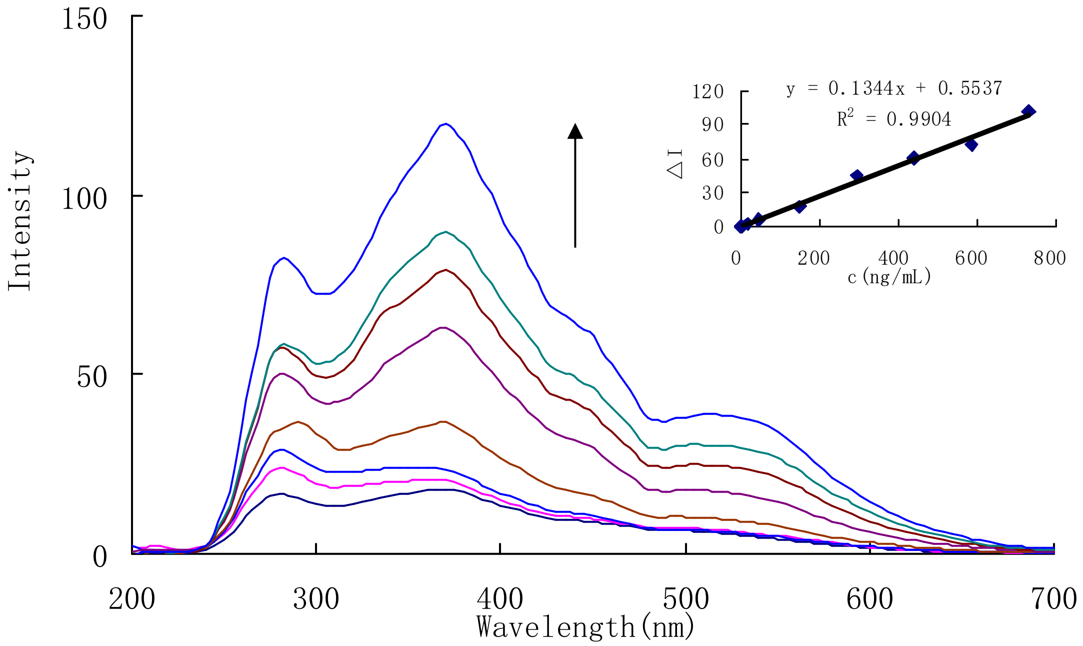

Figure S3. RRS spectra of CO-$HAuCl_4$-glucose nanocatalytic system. a: 0.24 mmol/L pH 5.2 HAc-NaA +5.6 mmol/Lglucose+0.56 mmol/L HCl+56.2 μg/mL $HAuCl_4$; b: a+14.6 ng/mL CO; c: a+43.9 ng/mL CO; d: a+146.2 ng/mL CO; e: a+292.5 ng/mL CO; f: a+438.7 ng/mL CO; g: a+584.9 ng/mL CO; h: a+731.2 ng/mL CO.

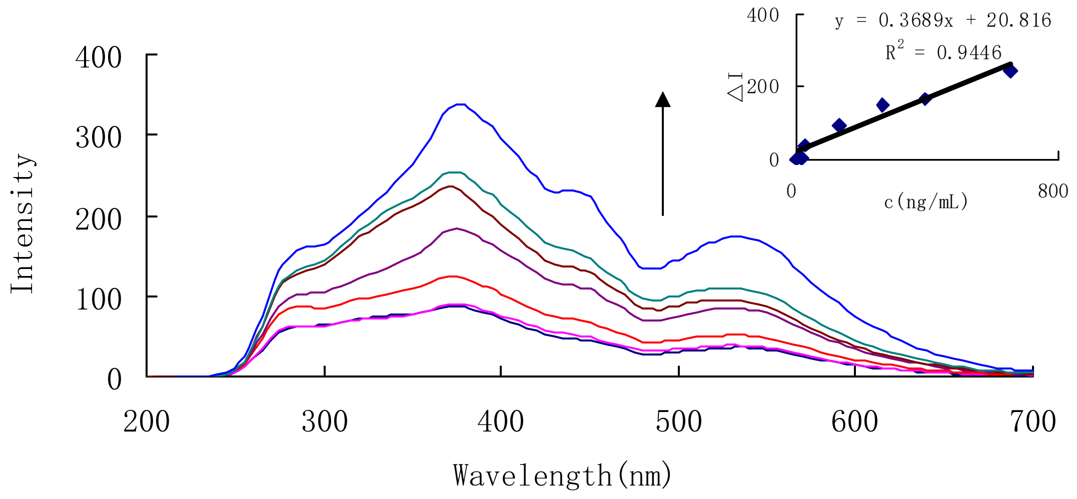

Figure S4. RRS spectra of CO-HAuCl4-glycerol system. a: 0.24 mmol/L pH 5.2 HAc-NaAc +1.5 % Glycerol+0.55 mmol/L HCl+50.2 μg/mL HAuCl4; b: a+13.1 ng/mL CO; c: a+26.2 ng/mL CO; d: a+130.8 ng/mL CO; e: a+261.6 ng/mL CO; f: a+392.4 ng/mL CO; g: a+654.0 ng/mL CO.

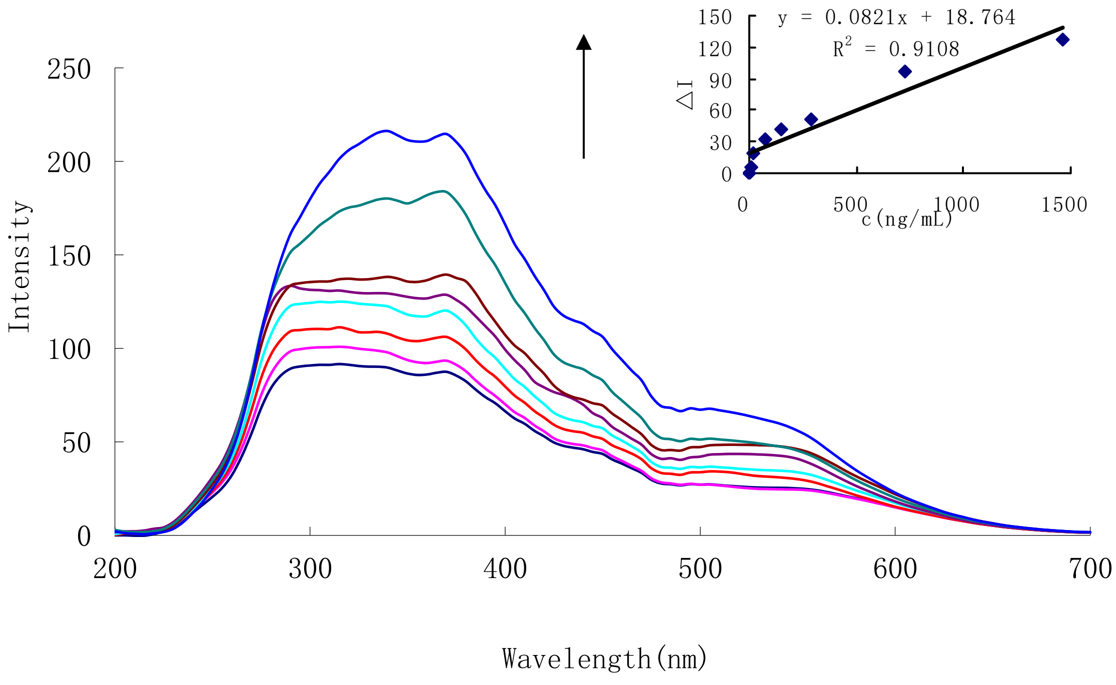

Figure S5. RRS spectra of CO-HAuCl4-citrate system. a: 0.24 mmol/L pH 5.2 HAc-NaAc +5.6 mmol/L citrate+0.61 mmol/L HCl+56.8 μg/mL HAuCl4; b: a+7.3 ng/mL CO; c: a+14.5 ng/mL CO; d: a+72.7 ng/mL CO; e: a+145.4 ng/mL CO; f: a+290.8 ng/mL CO; g: a+727.9 ng/mL CO; h: a+1454.2 ng/mL CO.

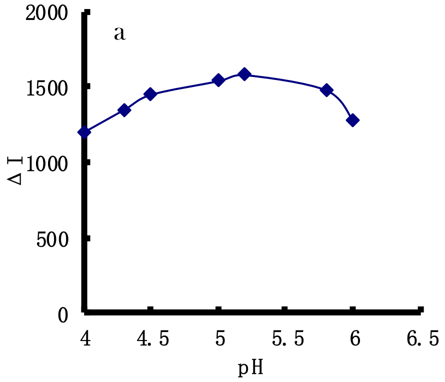
a
2000
1500
1000
500
0
ΔI
4
4.5
5
5.5
6
6.5
pH

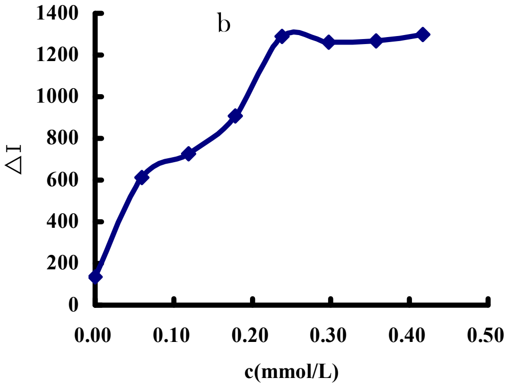
b
1400
1200
1000
800
600
400
200
0
ΔI
0.00
0.10
0.20
0.30
0.40
0.50
c(mmol/L)

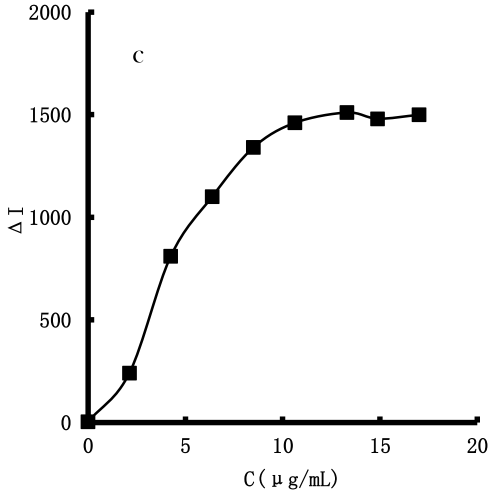
c
2000
1500
1000
500
0
ΔI
0
5
10
15
20
C(μg/mL)

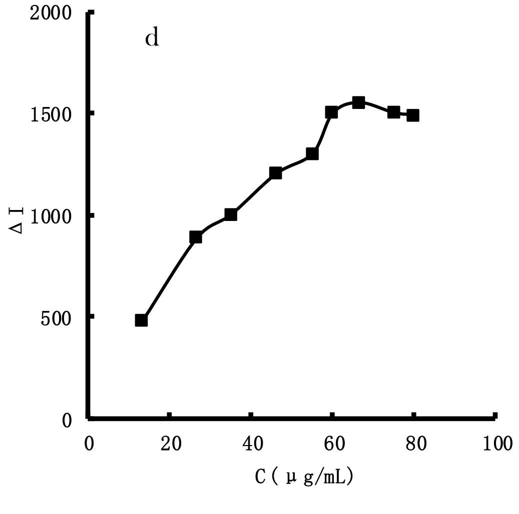
d
2000
1500
1000
500
0
ΔI
0
20
40
60
80
100
C(μg/mL)

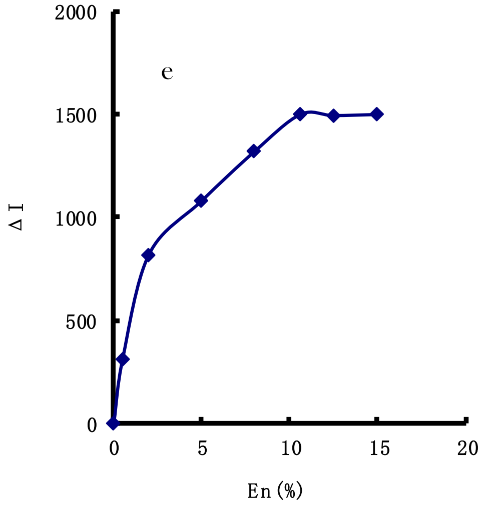
e
2000
1500
1000
500
0
ΔI
0
5
10
15
20
En(%)

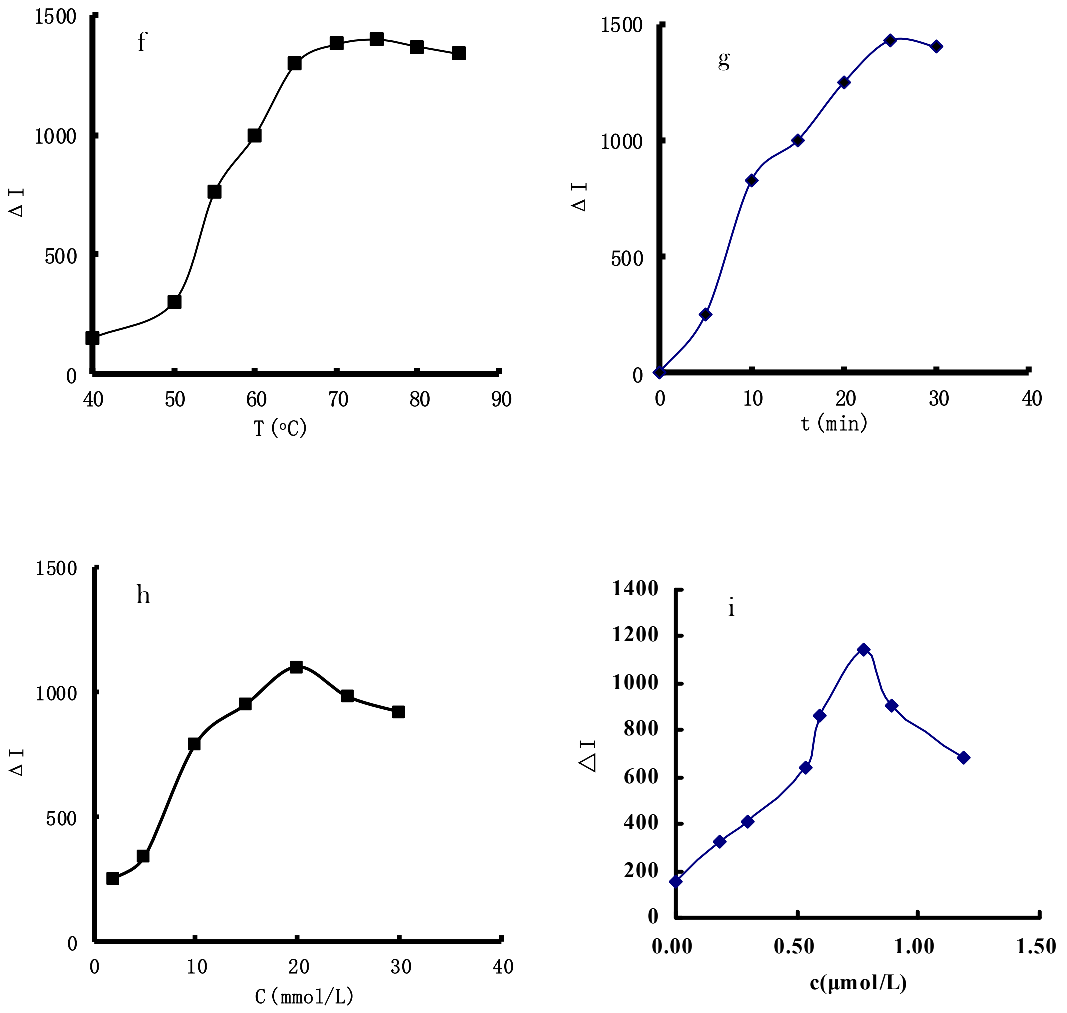

Figure S6. Effect of reaction conditions including reagent concentration, reaction temperature and time on the ΔI value. a: pH; b: buffer concentration; c: $HAuCl_4$ concentration for the CO-Au(III) system; d: $HAuCl_4$ concentration; e: En concentration; f: reaction temperature; g: reaction time; h: NaCl concentration; i: VBB concentration.

Table S1. Comparison of some reported methods for CO

Methods	T Principle	LR	DL	Comments	Ref.
Metal-based sensor	A device based on InP and GaS, which has two types both working in continuous-wave mode, generating light in single-mode emission at the desired wavelength of the CO absorption line.	-	2.0 μg/mL	fast, efficient, but high energy consumpti on, low sensitivity	[30]
	Stable and economically viable sensor modules for fast and efficient detection of CO based on (Ca-SnO2).	-	1.0 μg/mL		[32]
	A sensor based on the quartz enhanced photoacoustic spectroscopy (QEPAS), which was suitable in the infrared region, was established for CO determination.	-	-		[40]
	Establishing a practical sensing platform consisting of SnO_2 nanoparticle-decorated semiconducting single-walled carbon nanotubes for highly sensitive CO detection with fast response at room temperature.	-	1.0 μg/mL		[41]
	An amperometric electrochemical sensor based on LaGaO3 was demonstrated as highly sensitive to CO, which could be used for CO monitoring of exhaust gas from a small indoor combustion source.	-	-		[27]
	A trace-gas sensor for CO based on Pulsed Laser-Induced Photo-Acoustic Spectroscopy (PLIPAS) in conjunction with laser excitation wavelength of 213 nm was designed, fabricated and tested for the first time.	-	-		[42]
	Two palladium complexes were used as sensing materials to functionalize quartz crystal microbalances (QCM). And this sensor had been tested to determine carbon monoxide in air.	-	-		[43]
Spectrophoto metry	Using hemoglobin binding of dissolved CO to generating the two-component system of hemoglobin (Hb) and carboxyhemoglobin	0-0.15 μg/mL	23 ng/mL	high sensitivity, but complicat	[33]

	(HbCO).			ed	
	Based on the reduction of Pd (Ⅱ) by CO, the resulting monomer Pd reacts with iodate in the presence of chlorine to produce ICl_n^- type substances, which are easily extracted into benzene by ion pairing with pyronine G. Spectrophotometric determination of the extract can achieve the purpose of detecting CO.	20-400 μL/L	1 μL/L	Simple, but low sensitivity	[44]
Fourier infrared spectroscopy	Detecting the CO concentration and total pressure of the light bulb lumen by traditional Fourier transform infrared spectroscopy.	-	-	Complex operation, low accuracy	[35]
Head space-GC	Detecting the CO in aquatic products according the good linear relationship of CO concentration with peak area.	0.5-5.0 μg/mL	10 μg/kg	Quick and easy, low sensitivity	[36]
HS/GC/MS	CO can combine with hemoglobin to form carboxyhemoglobin (HbCO). The levels of CO and HbCO can be determinate according to the percentage of them.	-	0.1 mg/mL	Good repeatabili ty, high accuracy, but low sensitivity	[38]
SERS/RRS	The AuNPs generated by the catalytic reaction of CO-Au(III)-En has a strong RRS effect.	3.0-413 ng/mL	1 ng/mL	Simple and sensitive.	This method

Table S2. The effect of coexistence on the determination of CO

Coexistent substance	Tolerance (Times)	Relative error (%)	Coexistent substance	Tolerance (Times)	Relative error (%)
Zn^{2+}	200	-4.0	Mg^{2+}	100	3.0
K^{+}	200	5.3	Fe^{3+}	100	7.0
BrO_3^{-}	100	7.0	Glucose	200	-4.4
Na_2S	100	8.5	Pb^{2+}	100	-6.0
Na_2SO_3	50	6.3	Al^{3+}	100	-0. 4
Methanol	100	4.4	Cu^{2+}	100	4.5
Ni^{2+}	100	5.3	SeO_3^{2-}	100	4.7
Cr^{3+}	100	2.5	$Na_2S_2O_3$	80	4.8
Co^{2+}	100	-5.5	Formaldehyde	80	3.7
Ca^{2+}	200	-2.7	Mn^{2+}	80	3.6
